# Anti-inflammatory and cytotoxic evaluation of extracts from the flowering stage of *Celosia argentea*

**DOI:** 10.1186/s12906-020-02941-4

**Published:** 2020-05-24

**Authors:** Oluwafunmilayo Dorcas Adegbaju, Gloria Aderonke Otunola, Anthony Jide Afolayan

**Affiliations:** Medicinal Plants and Economic Development (MPED) Research Centre, Department of Botany, University of Fort Hare, Alice, 5700 South Africa

**Keywords:** *Celosia argentea*, Cell viability, Cytotoxicity, Flowering stage, Inflammation, Murine cells

## Abstract

**Background:**

This study was aimed at investigating the possible anti-inflammatory and cytotoxic effects of extracts from the flowering stage of *C. argentea.* This growth stage was chosen because of its high polyphenolic content and high antioxidant capacity.

**Methods:**

Anti-inflammatory potential of the aqueous, acetone and methanol extracts of *C. argentea* was evaluated through the inhibition of nitric oxide production (LPS-induced) on stimulated macrophages (RAW 264.7), while MTT assay was used to assess cell viability with Silymarin as standard. Cytotoxicity of the plant extracts was evaluated on murine preadipocyte cell line (3 T3-L1) using the image-based method of two DNA-binding dyes; Hoechst 33342 and propidium iodide (PI) with melphalan as standard.

**Results:**

Acetone extract exhibited moderate, dose-dependent anti-inflammatory activity with no significant toxicity to activated macrophages, however the aqueous and methanol extracts were unable to inhibit nitric oxide production at both trials. MTT assay and the toxicity assay revealed that the flowering stage extracts of *C. argentea* were not toxic to the RAW 264.7 macrophages and 3 T3-L1 cells at all the tested concentrations (0, 2, 50, 100 and 200 μg/mL).

**Conclusions:**

These findings corroborate the traditional use of *C. argentea* for painful inflammatory conditions and encourage its possible use as lead for the development of novel, non-toxic, anti-inflammatory agents.

## Background

Inflammation is a protective or healing response to tissue injury in the body. It is a process characterized by a complex cascade of reactions which can be prompted by several agents along with toxic compounds, microbes, impaired cells and accumulated exudates [1, 2]. However, uncontrolled and chronic inflammation can be deleterious to health. There is a growing body of knowledge that believes that chronic inflammation is a major accomplice in the pathogenesis of numerous modern chronic diseases such as obesity, hypertension, atherosclerosis, type 2 diabetes mellitus, Alzheimer’s, osteoarthritis, inflammatory bowel diseases, cardiovascular diseases and cancer [1]. Inflammation is usually associated with deregulation in the homeostatic mechanism of processes in human physiology [3, 4]. Persistent inflammation as a result of tissue dysfunction invariably triggers the release of macrophages.

Macrophages are large, specialized cells present in virtually all tissues [5]. They are essential cells of the immune system that are designed as inflammatory mediators in response to an infection or detection of damaged or dead cells They also play critical roles in immune regulation as they have the ability to recognize, engulf and destroy target cells as a response to a variety of cellular signals [6].. Due to the fact that these phagocytic cells are mostly short-lived and negative side-effects are associated with non-steroidal anti-inflammatory drugs (NSAIDs), it becomes pertinent to search for novel anti-inflammatory materials with minimal side- effects or none at all. Hence, the formulation of curatives from natural sources which can supplement the distinctive immune regulation of macrophages is vital to prevent existing and evolving chronic diseases. Studies of medicinal plants for development of modern drug have been very fruitful in the past few decades [7, 8] and have shown that plants possess a wide range of chemical compounds with biological activities [9, 10].

*Celosia argentea* (Amaranthaceae) also known as cock’s comb or quail grass is a herb of great nutritional and therapeutic importance. The seeds, leaves, flowers and roots are known for their folkloric uses in Chinese and India traditional medicine as an antidote for snake- bite, glandular swelling, uterine bleeding, leucorrhea and inflammation [11, 12]. The alcoholic extracts of the leaves of *C. argentea* have been reported to possess anti-diabetic property by lowering the body weight and blood glucose of diabetic rats (alloxan-induced) by 38.8% at 500 mg/kg body weight [13]. It has also been reported that the flavonoid fraction of *C. argentea* leaves possess anti-inflammatory activity on carrageenan-induced rat paw edema and cotton pellet-induced chronic inflammatory models at 10 mg/kg body weight [11]. Recently Malomo and Yakubu [14], reported that the aqueous extract of *C. argentea* attenuated cadmium-induced oxidative stress in Wistar rats at 400 mg/kg body weight.

Due to the pharmacological significance and low cost of plant derived substances compared to synthetic drugs, much attention has been drawn to them, especially for the discovery of unique and novel anti-cancer drugs as well as therapeutic agents for treating other viral infections [15]. Therefore, cytotoxicity screening of plant extracts intended for pharmaceutical production is an important initial step when investigating possible new therapies or developing new compounds for the treatment of an ailment. This will help to detect their possible cytotoxic and harmful effects.

The study is therefore aimed to investigate the anti-inflammatory potential and possible cytotoxicity of flowering stage extracts of *C. argentea.*

## Methods

### Plant source and study site

The plant material used for this study was harvested at the 7th week (flowering stage) of each trial from the glass house of the University of Fort Hare, Alice, South Africa. The geographical location of the study site lies at latitude 32^°^ 47′- 19^°^ 26′ S; longitude 26^°^ 50′- 42^°^ 306′E and altitude of 514.70 m. The plant was authenticated by Prof Cupido at the Giffen herbarium of University of Fort Hare and a voucher specimen (Ade/med/2017/01) was deposited at herbarium for reference.

### Preparation of crude extracts

After harvest, aerial parts (leaves, stem and flowers) of the plant were rinsed with de-ionized water, oven-dried for 72 h at 40 °C and pulverized with an industrial electric blender (Hamilton Beach, HBF500s series, Canada). 200 g of the ground sample individually in 1.5 L of distilled water, acetone or methanol was agitated constantly for 72 h using a shaker (Gallenkamp incubator orbital shaker). Filtered solution from the aqueous extract was concentrated to dryness in a freeze dryer, while the solvent extracts were vaporized to dryness in a rotary evaporator at their respective boiling points. The dried extracts were stored at 4 °C in a refrigerator until needed.

#### Chemicals and cells

All the chemicals and reagents used including glucose, pyruvate, dimethyl sulfoxide (DMSO), Griess reagent, Hoechst 33342, Propidium iodide, Cell lines and culture materials including 10% fetal calf serum, Melphalan, DMEM (PI), 3 T3-L1; RAW 264.7 cell-lines and RPMI medium were sourced from Sigma-Aldrich® (Johannesburg, South Africa) and Hyclone® (Thermo Fisher, Logan, UT, USA).

#### Anti-inflammatory assay

##### Cell culture

RAW 264.7 macrophages were cultured in the appropriate medium containing 10% fetal calf serum (Roswell Park Memorial Institute medium (RPMI) and were inoculated in 96-well plates (at a density of 1 × 10^5^ cells per well), then allowed to incubate overnight in a humidified incubator at 37 °C and 5% CO_2_ for 24 h. The medium was replaced with aqueous, acetone and methanol extracts of *C. argentea* at concentrations ranging from 0 to 200 μg/mL obtained using serial dilution, with silymarin as positive control. A volume of 1 μg/ml of lipopolysaccharide was added to the test samples and incubated at 37 °C for 18 h. Thereafter, cell viability was assessed by adding 50 μl of Griess reagent and medium into each well of another 96-well plate. It was then allowed to stand for 10 min at 27 °C. Absorbance was measured at 540 nm and the concentration of nitrate released into the culture medium was calculated from the slope of the standard curve as.

##### MTT assay

Simultaneous evaluation of cell viability was determined colorimetrically by adding 100 μL culture medium containing 0.5 mg/mL MTT to the remaining cells and allowed to stand at 37 °C for 1 h. The supernatants were decanted and the formazan precipitate in each well was dissolved in 100 μl DMSO. Cell viability was assessed by measuring the absorbance at 560 nm.

##### Cytotoxicity assay

Dimethyl sulphoxide (DMSO) was used to reconstitute the extracts to give a final concentration of 100 mg/mL. The resultant solution was sonicated and the murine preadipocyte cell line (3 T3-L1), was cultured in a medium of DMEM with low glucose and pyruvate, supplemented with 10% fetal calf serum. The cells were seeded into 96-well microtiter plates at a density of 3000/well and volume of 100 μL per well. This was incubated at 37 °C in 5% CO_2_ and 100% relative humidity for 24 h, before the addition of test compounds to allow for cell attachment. 100 μL of the aqueous, acetone and methanol extracts diluted at four concentrations (0, 50, 100 and 200 μg/mL) was added to each well. The cells were incubated at 37 °C in a humidified 5% CO_2_ for 48 h. The treatment medium was removed from the wells and substituted with 100 μL of Hoechst 33342 nuclear dye for 10 min at 25 °C. The cells were stained with 100 μg/mL propidium iodide (PI) for viewing and counting the number of dead cells.

### Data quantification

All data were expressed as means ± standard deviation (SD). Means were accepted as significantly different when data showed (*P* < 0.05), using one-way analysis of variance (ANOVA) and Fischer’s LSD with the aid of GENSTAT 8 software.

## Results

### Inhibition of nitric oxide production in RAW 264.7 by extracts of the flowering stage of *C. argentea*

**Fig. 1:**
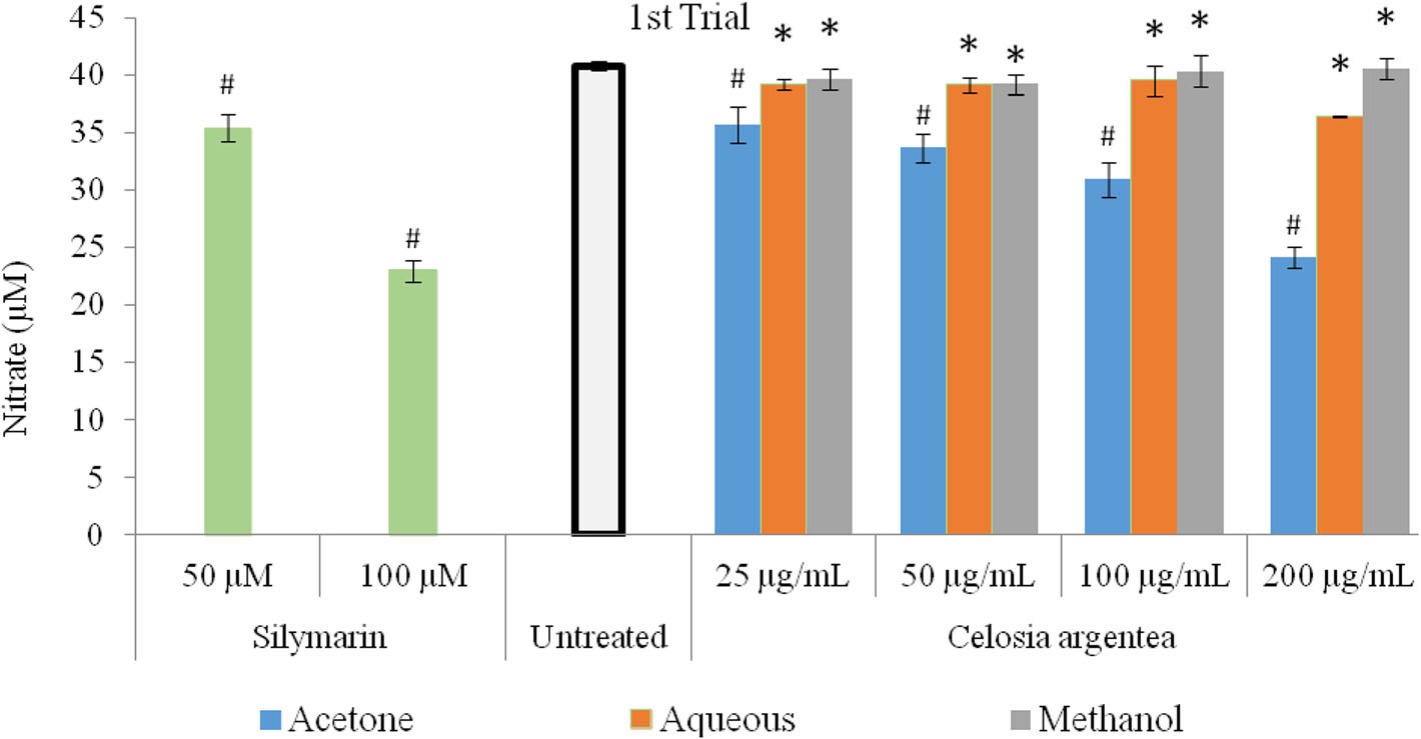
Inhibition of LPS-induced nitric oxide production in RAW 264.7 cells by extracts of *C. argentea*. Data are mean ± standard deviation *n* = 3. Silymarin was used as positive control to illustrate inhibition of LPS induced nitrate levels * indicates no significant difference from the untreated control. (First trial)

**Fig. 2:**
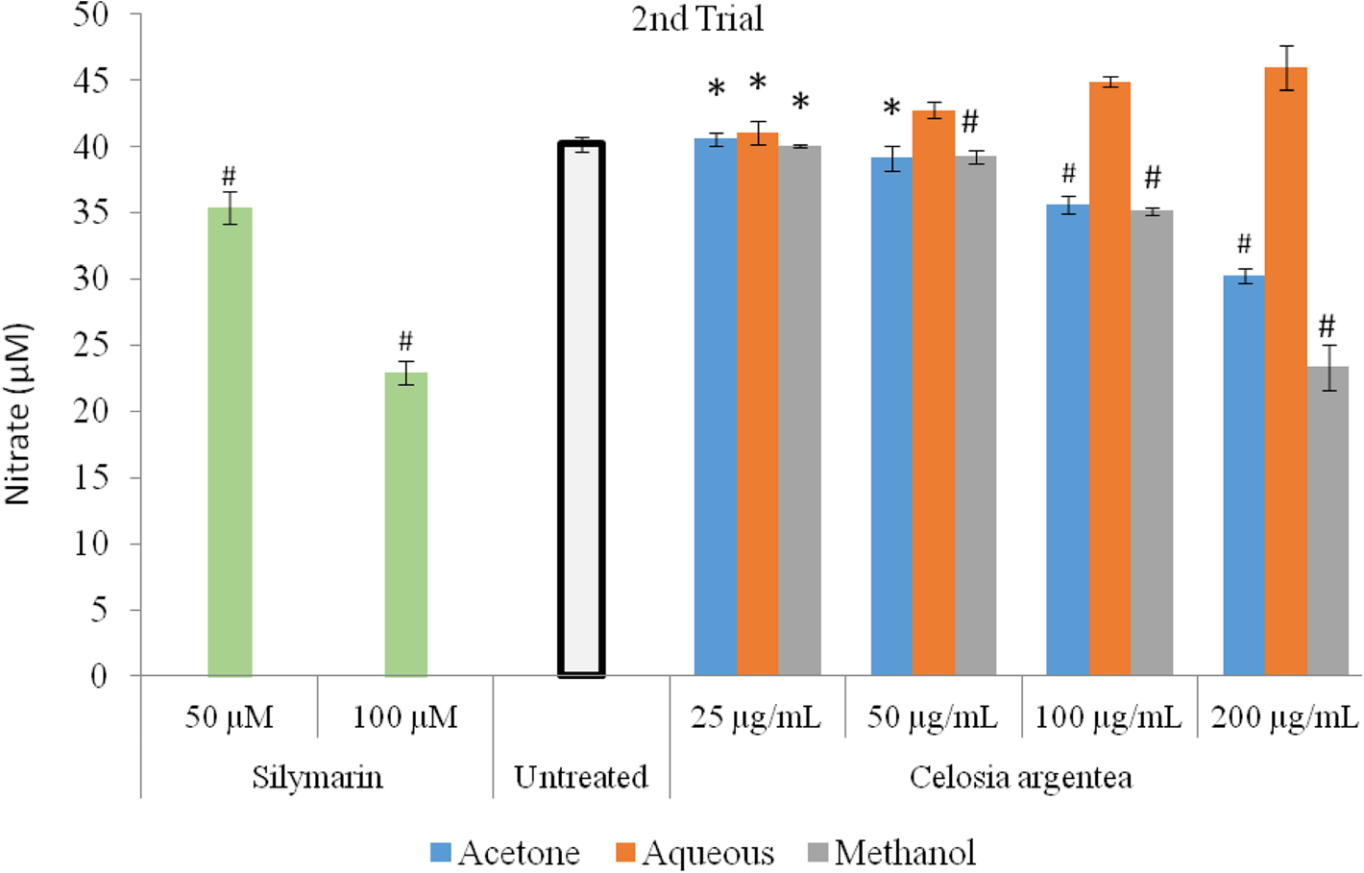
Inhibition of LPS-induced nitric oxide production in RAW 264.7 cells by extracts of *C. argentea*. Data are mean ± SD, *n* = 3. Silymarin was used as positive control to illustrate inhibition of LPS induced nitrate levels, * indicates no significant difference from the untreated control. (Second trial)

**Fig. 3:**
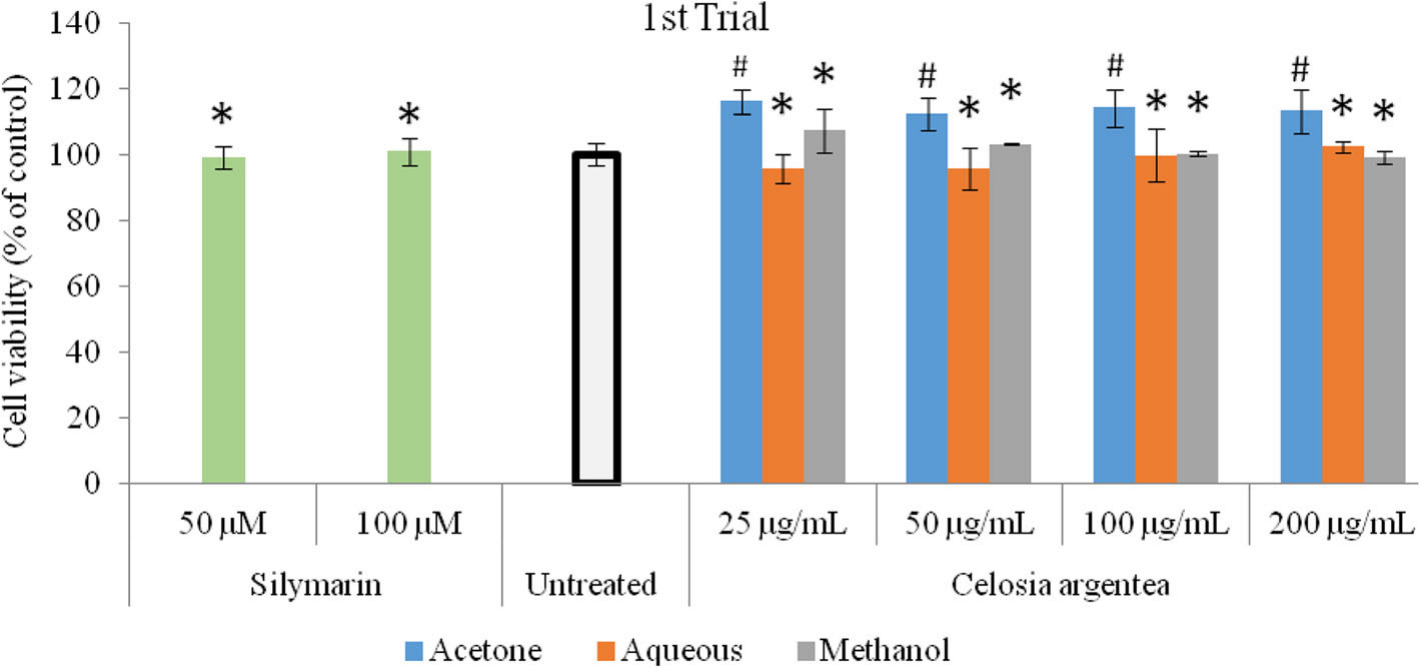
Cell viability of RAW 264.7 macrophages exposed to flowering stage extracts of *C. argentea.* Data are mean ± SD, *n* = 3. Silymarin was used as positive control. * indicates no significant difference from the untreated control; # indicates significantly higher than the untreated control. (First trial)

**Fig. 4:**
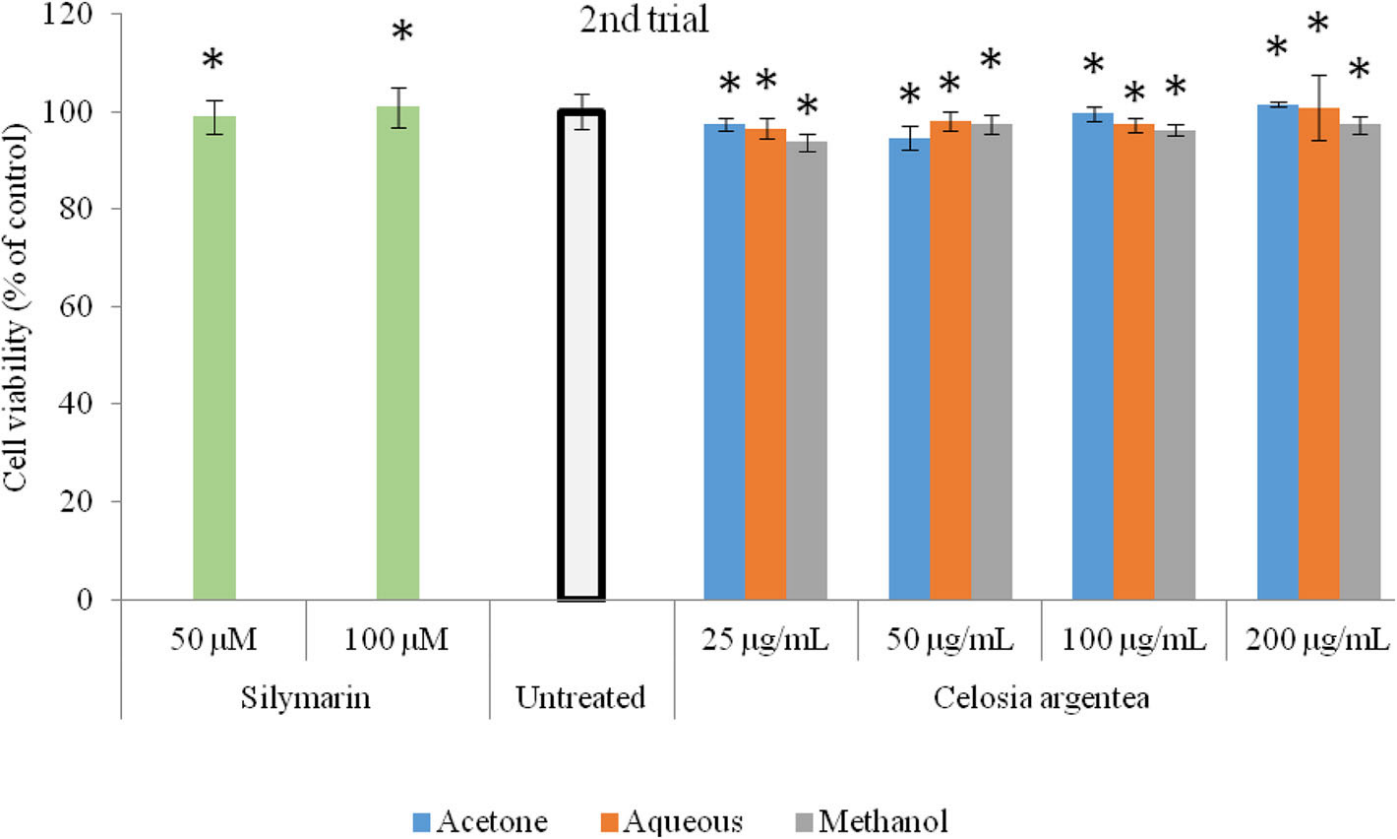
Cell viability of RAW 264.7 macrophages exposed to flowering stage extracts of *C. argentea*. (Second trial). Data are mean ± SD, *n* = 3. Silymarin was used as positive control. *indicates no significant difference from the untreated control; #indicates significantly higher than the untreated control. (Second trial)

The inhibitory activity of flowering stage extracts of *Celosia argentea* against nitric oxide production in RAW 264.7 macrophages (LPS-induced) and corresponding cell viability are presented in Figs. 1 and 2. The MTT assay was used to verify if observed inhibitions of nitric oxide production in the cell were caused by cell death or activity of the test compounds (Figs. 3 and 4). The first trial, revealed that aqueous and methanol extracts of the flowering stage of *C. argentea* did not suppress the production of NO in the cells compared to the untreated control. The acetone extracts however, suppressed LPS-enchanced NO expression in a dose-dependent manner. For the second trial, both the acetone and methanol extracts exhibited a dose-dependent anti-inflammatory activity by inhibiting NO production in the cells, which is comparable to the positive control. While the acetone showed better reduction of NO production in the first trial, the methanol extract of the second trial had the highest inhibitory power against NO production in the macrophages.

### Cell viability

The cell viability evaluation revealed that the extracts along with the positive control were significantly (*p* < 0.05) non-toxic to the macrophages and that the observed inhibitory action of the extracts was not due to cell damage. In comparison to the untreated control, Cell proliferation was observed in the acetone extract of the first trial (Figs. 3 and 4).

### Response of 3 T3-L1 cells to flowering stage extracts of *C. argentea*

**Fig. 5:**
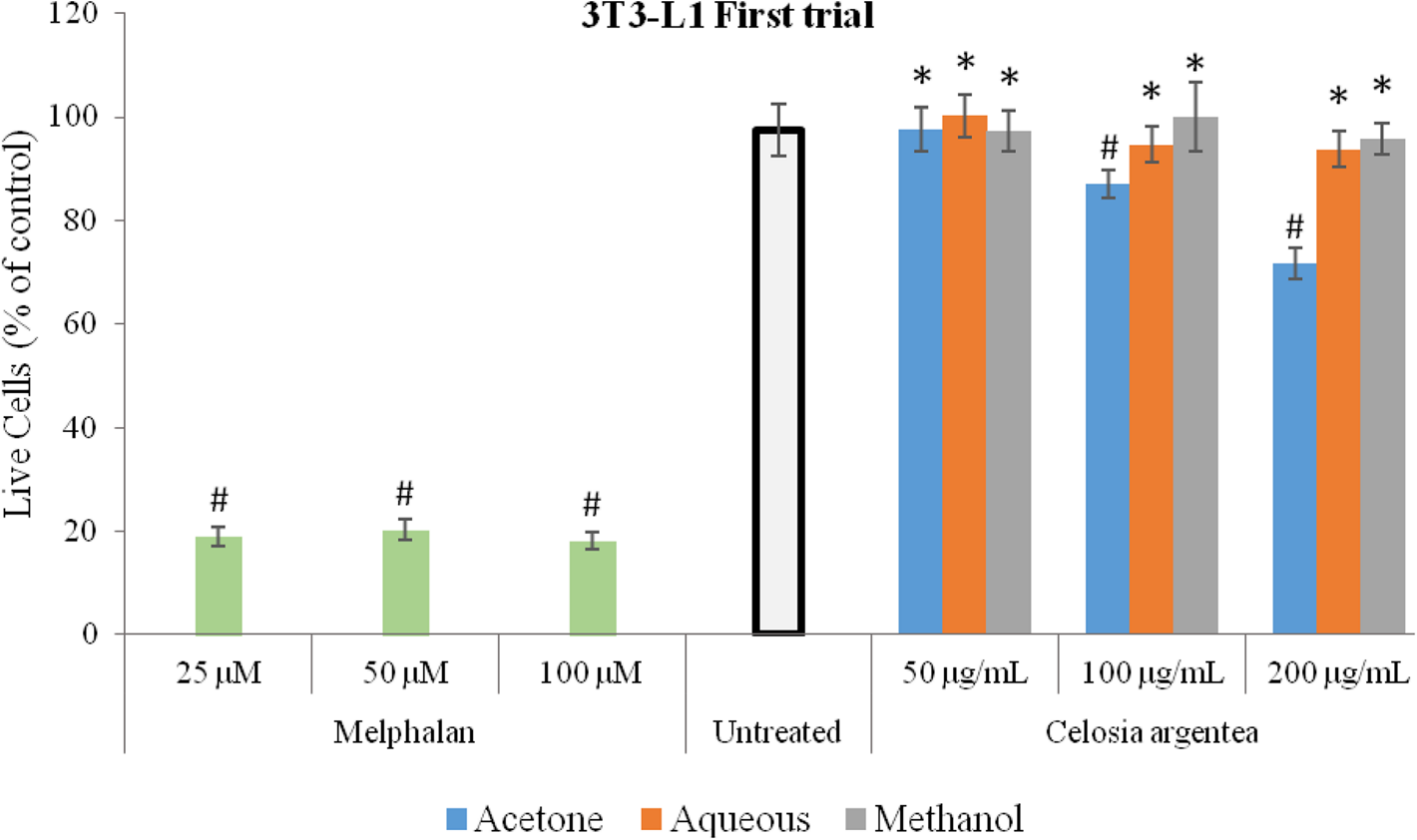
Cytotoxic response of 3 T3-L1 cells to extracts of flowering stage of *C. argentea* at first trial. Error bars indicate SD, *n* = 4 from a single experiment. * indicates no significant difference from the untreated control; #indicates significantly lower than the untreated control

**Fig. 6:**
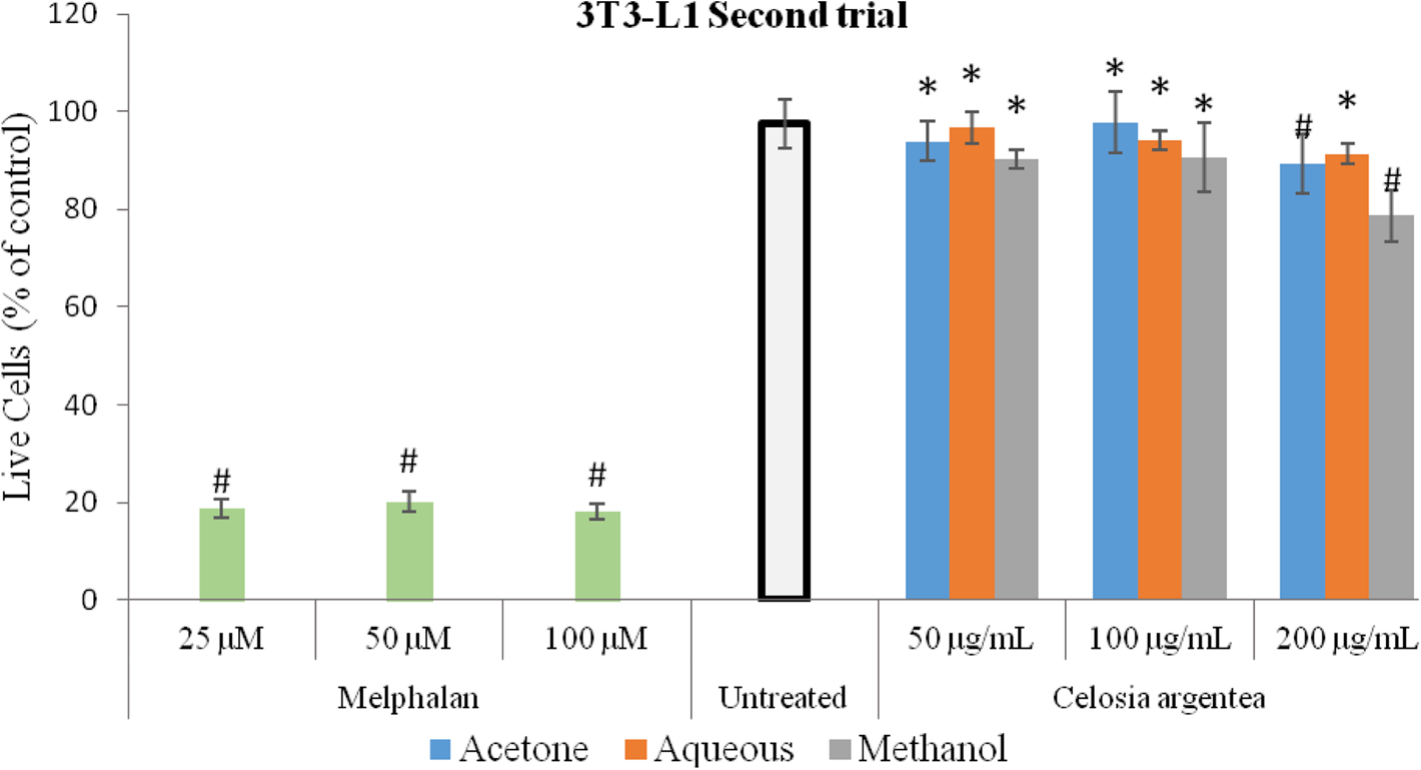
Cytotoxic response of 3 T3-L1 to flowering stage extracts of *C. argentea* at second trial. Error bars indicate SD, *n* = 4 from a single experiment. * indicates no significant difference compared tothe untreated control; # indicates significantly lower than the untreated control

The effect of extracts of the flowering stage of *C. argentea* on murine preadipocyte cells (3 T3-L1) is depicted in Figs. 5 and 6. For the first trial, effect of the aqueous and methanol extracts was not significantly different from the untreated control at all concentrations. A dose-dependent significant difference (p<0.05) at 100 and 200 μg/mL was however observed with the acetone extract compared to the untreated control, indicating low toxicity at 200 μg/mL. For the second trial, compared to the untreated control, significant cell reduction was observed for the acetone and methanol extracts at the highest concentration, with the methanol extracts showing low cytotoxicity on the 3 T3-L1 cells. The positive control (Melphalan) however was significantly toxic to the cells at all concentrations.

## Discussion

Nitric oxide (NO) is a key inflammatory mediator and excessive production due to the reaction of free radicals within biological systems, can result in various diseases such as cardiovascular diseases, cancer and atherosclerosis [16]. Since most diseases are associated with the disruption of homeostasis and the production of excess NO, hence, lowering its production may be of healing advantage in various diseases induced by pathological levels of NO [17].

The acetone and methanol extracts of *C. argentea* moderately inhibited nitric oxide production in the macrophages in a dose-dependent manner. Such dose dependent inhibitory activity of *C. argentea* extracts observed in this study was similar to the findings of Malomo and Yakubu [15], who reported increased reducing power activity of *C. argentea* extract with increasing concentration. This also corroborates the findings of Wu et al. [18], who reported that four saponins isolated from *Celosia argentea* seeds (Celosin E, F, G and cristatain) had inhibitory action against nitric oxide production. The moderate inhibitory activity observed here indicates that *C. argentea* extracts could act as protective mediators against free radicals and as a regulatory agent with homeostatic activities.

Non-steroidal anti-inflammatory drugs (NSAIDs) are a wide group of cyclooxygenase (COX) inhibitors with similar therapeutic actions [18], whose interaction with cell membranes have been shown to initiate many inflammatory phenomena and toxic side effects [19, 20]. MTT assay revealed that none of the extracts from both trials at all concentrations evaluated in this study was cytotoxic. The significantly higher cell volume reported for the acetone extract indicate that it supports cell proliferation, which could be a positive development in wound healing, tissue repair and and aging [20]. Thus, the increased cellular proliferation observed in this study, suggest that the *C. argentea* extracts from the flowering stage could be used therapeutically for wounds, injury, repair and reversal of tissue damage.

Therapies from natural products and herbal medicines have shown promising potential as relevant complementary and alternative treatments against several aliments with the efficacy of a good number of herbal products clearly established [21]. However, the degree of use of herbal medicines does not correspond to the extent at which their safety is considered [22]. According to Vijayarathna and Sasidharan [23], any crude plant extracts at concentration of 20 μg/mL and below, that produces 50% of cell death within 72 h in vitro is cytotoxic. Worthy of note is the cytotoxic response of the 3 T3-L1 cells to all the extracts, as the percentage of dead cells was not up to 50% at all the concentrations tested. This implies therefore, that all extracts from the flowering stage of *C. argentea* are not toxic. This corroborates the reports of Rub et al [24], who stated that the methanolic extract of *C. argentea* showed no significant toxicity towards SiHa and MCF-7 cell lines. The non- toxic potential of all the extracts in this study corroborates the traditional use of the plant as vegetable and medicine; and indicates that *C. argentea* could be safely used as an anti-inflammatory agent with no side-effects.

## Conclusions

This study has shown that the flowering stage extracts of *C. argentea* possess moderate inhibitory activity against the production of nitric oxide (Lipopolysaccharide-induced) in RAW 264.7 cells and corroborates the traditional use of the plant for painful inflammatory conditions, while encouraging its use for the development of non-toxic novel anti-inflammatory agents.

## Data Availability

The datasets used and/or analyzed during the current study are available from the corresponding author on reasonable request.

## Abbreviations

ANOVA: Analysis of variance
DMEM: Dulbecco’s Modified Eagle Medium
DMSO: Dimethyl sulphoxide
DNA: Deoxyribonucleic acid
LSD: Least significant difference
LPS: Lipopolysaccharide
MCF-7: Michigan Cancer Foundation-7
MTT: 3-(4,5-Dimethylthiazol-2-Yl)-2,5-Diphenyltetrazolium Bromide)
NSAID: Non-steroidal anti-inflammatory drugs
PI: Propidium iodide
RPMI: Roswell Park Memorial Institute
SiHa: Human cervical cell line
